# Elucidating emotional closeness within the Theory of Health-Related Family Quality of Life: evidence from breast cancer survivors

**DOI:** 10.1186/s13104-019-4354-5

**Published:** 2019-06-03

**Authors:** M. Elise Radina, Briana L. Deer, Rachael A. Herriman, Anjana Jagpal, Mallory M. Dodd, Kaylie L. Kawamura, Lindsay C. Clark

**Affiliations:** 1 Department of Family Science & Social Work, Miami University, 101 McGuffey Hall, 210 E. Spring Street, Oxford, OH 45056 USA; 2 Kansas City University of Medicine and Biosciences, Kansas City, USA; 3 Boonshoft School of Medicine, Wright State University, Dayton, USA; 4 Department of Psychology, DePaul University, Chicago, USA; 5 Thermo Fisher Scientific, Waltham, USA; 6 Relevate Health Group, Cincinnati, USA; 7 College of Optometry, University of Missouri-St. Louis, St. Louis, USA

**Keywords:** Quality of life, Family, Communication, Social Support, Theory, Qualitative methodology

## Abstract

**Objectives:**

Due to the increasing survivorship of breast cancer, survivor’s view of their families through the process of diagnosis and treatment is essential. The Theory of Health-related Family Quality of Life (HRFQoL) guided this exploration of the ways in which breast cancer impacts family life. In this study, HRFQoL was used to explore breast cancer survivors’ perceptions of the theory’s sub-concepts of psychological and/or affectional closeness, family communication, and social support. The guiding research question was: In what ways do breast cancer survivors describe their experiences regarding changes in emotional closeness among family members following their breast cancer diagnosis? Participants (N = 22) were interviewed to discuss their experiences with breast cancer, family quality of life, decision-making, basic health information, and personal coping. Data were analyzed using NVivo 9 to conduct thematic analysis and consensual qualitative data analysis.

**Results:**

Diagnosis and treatment of breast cancer improved the majority of participants’ HRFQoL. Participants who reported positive perceptions prior to diagnosis also reported positive perceptions after diagnosis. These findings elucidate the HRFQoL theory and contribute to understanding how breast cancer impacts family life.

**Electronic supplementary material:**

The online version of this article (10.1186/s13104-019-4354-5) contains supplementary material, which is available to authorized users.

## Introduction

The number of invasive and in situ case of breast cancer continues to rise while survival rates remains high with 3.5 million women currently living as breast cancer survivors [1]. Thus, research is increasingly about survivorship. Because breast cancer impacts the entire family, not just the patient/survivor [8] it is essential to gain understanding of family quality of life for the effected families as quality of life can be affected for the better and for the worse [5]. Radina [7] Theory of Health-Related Family Quality of Life (HR-FQoL) guided this study that explored breast cancer survivors’ perceptions of how their diagnosis, treatment, and survivorship experiences impacted their HR-FQoL.

## Main text

### Methods

After receiving Institutional Review Board approval (Miami University #05-060), participants were recruited using email listservs and breast cancer related organizations. All participants (N = 22) identified as Caucasian, female, and having at least a high school level education. Participants were married (n = 15), previously married (n = 4), engaged (n = 2), or unmarried (n = 1). Eighteen participants had children. Information regarding cancer staging was not collected as a part of this study (Table 1).

**Table 1:** Participant demographics

	Mean (range)
Age	57.8 years (42–87 years)
Length of relationship	30.94 years (0.67–63 years)
Education	16.1 years (12–22 years)

Recruited participants, having indicated their informed consent, were interviewed in person or over the phone to discuss their experience with breast cancer, family quality of life, decision-making, basic health information, and personal coping (Additional file 1: Appendix A: Interview Guide).

The present study focused on the portions of interview transcripts that contributed to elucidating the overarching concept of emotional closeness. The guiding research question was: In what ways do breast cancer survivors describe their experiences regarding changes in emotional closeness among family members following their breast cancer diagnosis? In order to explore this question data analysis focused on [7].

Data were analyzed using qualitative methodology that included a codebook and consensual qualitative data analysis [4]. Undergraduate research assistants used a codebook guided by Radina [7] theory of HRFQoL to code the interview transcripts. That is, prior to data coding, codes for the codebook were created that focused on the theory’s sub-concepts of psychological and/or affectional closeness, family communication, and social support. For more information on these sub-concepts, please see Radina [7]. For the purposes of the Research Note format, which limits word count, these findings are presented quantitatively and with select individual quotes as appropriate.

### Results

#### Emotional satisfaction

Of those who described emotional satisfaction, 15 participants mentioned their perceived satisfaction both before and after diagnosis. Of these, all but one described positively their satisfaction both prior to and after diagnosis. Fifteen participants mentioned satisfaction with how their family met their personal needs prior to and after diagnosis. Three out of 22 participants mentioned satisfaction only after diagnosis. Of the three, two were not satisfied. Due to the fact that these three participants did not mention emotional closeness prior to diagnosis, no change over time can be determined.

#### Affectional–psychological closeness

The concept of affectional–psychological closeness is defined as feelings of either positive or negative psychological or affectional closeness towards and/or between family members as well as such feelings towards the family. Out of 22 participants, a total of 19 talked about affectional–psychological closeness. Three did not talk about affectional–psychological closeness at all, even when prompted. Of the 22, 10 participants talked about affectional–psychological closeness both before and after diagnosis. Eight out of these 10 participants expressed a positive relationship before and after diagnosis. Amy, the only participant to describe affectional–psychological closeness as having changed over time, saw the relationships as positive and negative prior to diagnosis, and only positive after diagnosis. Only one participant experienced no change in affectional–psychological closeness over time.

Seven of the 19 participants who talked about affectional–psychological closeness only mentioned this it when they described their family relationships after diagnosis. Of the seven, six participants talked positively about closeness. One participant spoke negatively about the affectional–psychological closeness. Due to the fact that none of these seven participants described affectional–psychological closeness prior to diagnosis, no change over time of this concept can be determined.

#### Feelings about communication

Overall all of the 22 participants mentioned feelings about communication. There were only three participants who mentioned feelings about communication prior to diagnosis. Even though all participants talked about feelings regarding communication, total instances were examined to determine potential themes: “Can Handle It”, “Emotional Distance” and “Uncomfortable with Communication.”

##### Can handle it

Participants suggested feeling a greater sense of that they could handle the cancer through the conversations that were held with others. This theme was found when examining positive responses regarding feelings about communication. Of the eight total instances where the participants reported feeling positive about the communication with family as a whole, this theme of “Can Handle It” was present in six of them.

##### Emotional distance

Though there were eight instances of positive feelings about the communication, there was almost an equal amount (n = 7) of adverse feelings about the communication. Among these instances where the participant felt unsatisfied with the communication (n = 3), negative feelings corresponded with emotional distance within the family.

##### Uncomfortable with communication

Within these seven instances of adverse feelings about communication, some participants felt uncomfortable talking about the situation. Regardless of how participants felt about discussing the cancer, they mostly felt hesitant because they perceived that others felt uncomfortable hearing about the cancer.

#### Support among family

Support among family includes references to social support, or lack thereof, provided by, or received from, other family members. When examining support among family, two themes were evident: “Physical Presence of the Family” and “Communication.”

##### Physical presence of the family

The physical presence of family members was a major theme in emotional support among family after diagnosis. Presence of family members occurred at the hospital (e.g., post-surgery, during chemotherapy) and at home. Participants reported 19 positive instances of increased family presence after the diagnosis. Participants not only talked about general family presence, but also the importance of having their children around. This presence contributed to emotional support among the family. Even with distance, participants still experienced family presence after diagnosis. Ruth (78, white, married, mother of three) stated that her family members were “Still supportive…the [adult–child] from Indiana, I fly out there and visit with them. And our daughter in Ames comes home regularly, she’ll just stop up for the day sometimes.” Even though there was a considerable distance between Ruth and her family members, she still received emotional support through a physical presence.

##### Family communication

Communication was seen before and after diagnosis through phone calls and open dialogue. This positive emotional support was shown in nine instances where there was one instance before the diagnosis and eight instances after. There were a total of four instances where participants did not feel emotional support through communication. One instance occurred before and three occurred after diagnosis. Tracy talked about discussions with her husband, “He was mostly off on another planet so it wasn’t, uh, extremely helpful, you know, it was more operational, well [inaudible]—‘how are we going to handle this? How are we going to handle that?’”.

##### Support for family

Support for family is instances in which the family (or specific family members) did or did not receive social support from those outside the family. Emotional support for the family came from predominately four sources healthcare providers, bosses and co-workers, friends, and spiritual communities.

##### Support from healthcare providers

In a total of 19 instances, participants mentioned emotional support for the family by healthcare providers. Of these, 15 expressed positive experiences after breast cancer diagnosis. Many described a genuine concern from the doctor. Participants described in-depth discussions as healthcare providers took time to consider every possibility in treatment. When a doctor included the family, participants expressed a feeling of support. When a doctor did not include the family, participants did not feel support from the doctor.

##### Support from employers and coworkers

Of the 11 instances that comprised this theme, nine were positive forms of emotional support. Although one instance involved a lack of connection with coworkers before the diagnosis, the others indicated increased support from coworkers. When coworkers had a sense of understanding regarding breast cancer they were able to provide emotional support in the workplace.

##### Support from friends

There were six instances where participants experienced a loss of friends after diagnosis attributed to the friends’ inability to cope, inability to handle the situation, or inability to follow through with offered support. Some participants experienced positive support from friends after diagnosis who noted that conversations about the cancer and anything either of them were concerned about in general helped both the friends and the participant cope.

##### Spiritual support

There were 12 instances where participants talked about spiritual support. Although there were two instances where participants did not feel spiritual support after the diagnosis, there were 10 instances where participants felt strong spiritual support. This spiritual support was seen through members of the church, prayers and discussion groups.

## Discussion

This study examined qualitative data generated from breast cancer survivors in order to elucidate the concepts of affectional–psychological closeness, feelings about family communication, support among the family, and support for the family within the Health-related Family Quality of Life Theory [7]. These findings suggest that some breast cancer survivors may experience changes in their feelings of emotional satisfaction within the family and their sense of affectional–psychological closeness. Similar to that reported by Biffi and Mamede [2] and Chou et al. [3], these findings suggest that using open communication allowed families to voice concerns and provide comfort for the survivor and for family members. Health-care providers should make efforts to provide families with opportunities and support to engage in this open communication. That being said, communication among family members was not always beneficial to participants; only half of the participants felt empowered by conversations with family. Similar Mallinger et al. [6] findings, negative feelings about communication usually corresponded with emotional distance within the relationship or discomfort in speaking about the diagnosis with particular family members. Thus, in providing opportunities and support for family communication, health-care providers should assess the patients relationship history and provide referrals to marriage and family therapists, family life educators, social workers, and other who may provide skills and support in navigating difficult family communication patterns.

Support for family was one of the major factors influencing participants’ perceptions of emotional support. Healthcare providers provided emotional support for the family by providing information and promoting inclusivity of family in treatment and decision-making. This type of support allowed families to feel more secure in decisions regarding breast cancer. Spiritual groups provided emotional support that allowed the family to feel a continuation of security and comfort established by the healthcare providers. Coworkers provided emotional support for the family by demonstrating humility and understanding. After diagnosis, participants noted that while they received support from some friends, they lost support and friendships in others. Survivors who did not experience a loss of friends noted that having support helped both the friends and the survivor cope; that they “could handle it.” These findings suggest that providing support for the patient alone may not be sufficient. These participants were most impacted by the perceived support their families received. This may be an artifact of women’s gender roles as care takers within family life. Regardless, patients and their families should be referred to those organizations and services that can provide a family-centered level of support.

## Limitations

This qualitative study focused on the individual reports of female breast cancer survivors regarding their perceptions of their family quality of life. While the choice of an all female participant group is appropriate given breast cancer’s limited prevalence among males, future research should explore the ways in which male survivors perceive their health-related family quality of life in the context breast cancer. Also, one of the chief challenges of family research is the approach to gaining family-level understanding of family dynamics. These data only offer the participant’s point of view. The sample size for this study is generally considered appropriate for descriptive, qualitative studies. Certainly, the ability to make generalizations beyond these data is limited. Future research should incorporate other family members’ points of view and/or collect family-level data using dyad or group interview formats. These participants were predominately well-educated, white, and middle-aged or older. Certainly, future research should explicitly seek out a diversity of perspectives.

## Additional file

**Additional file 1.** Appendix A: Interview Guide.

## Data Availability

Please contact author regarding data requests.

## Abbreviation

HRFQoL: the Theory of Health-related Family Quality of Life
