# Supplementary material for: Elucidating emotional closeness within the Theory of Health-Related Family Quality of Life: evidence from breast cancer survivors
Source: BMC Res Notes. 2019 Jun 3;12:312. doi: 10.1186/s13104-019-4354-5 (PMC6545688; doi:10.1186/s13104-019-4354-5)
Supplement: Supplementary file 1 — Additional file 1. Appendix A: Interview Guide. [file 13104_2019_4354_MOESM1_ESM.docx]

Appendix A: Interview Guide

## Your Experience

## To begin, tell me a little bit about your breast cancer experience [staging, treatment, age at diagnosis, current health status, troubles/triumphs].

How had your health been up to the time your breast cancer was diagnosed?

How are things going for you, so far?

- Doing very well
- Having mild-to-moderate difficulty
- Doing very poorly

## Family/Relationship Quality of Life before Breast Cancer Diagnosis

Describe for me what your family/relationship quality of life was like before your diagnosis of breast cancer.

What was your perception of the level of support received by family members from each other?

What was your perception of the level of support received by family members from people outside the family?

What were areas of challenge for your family?

How did your family deal with these challenges?

What did you perceive as your family’s/relationship’s strengths?

Describe how satisfied you were with your relationship with other family members.

What responsibilities did particular family members take on?

To what degree were you satisfied with the way your family met your personal needs?

**Family/Relationship Quality of Life AFTER Breast Cancer Diagnosis**

Since your diagnosis, describe for me what your family’s quality of life is like now.

What is your current perception of the level of support received by family members from each other?

What is your current perception of the level of support received by family members from people outside the family?

What are areas of challenge for your family?

How does your family deal with these challenges?

What do you perceive as your family’s strengths?

Describe how satisfied you are with your relationship with other family members.

What responsibilities do particular family members currently take on?

To what degree are you currently satisfied with the way your family meets your personal needs?

**Your Coping**

Did relationships change after the diagnosis between you and friends, coworkers, extended family members, etc?

In what ways did your behavior among friends and coworkers and extended family change after the diagnosis?

Who did you turn to for support after the diagnosis?

How, if at all, was this person/were these people different from your previous support networks?

Were your treatment, initial diagnosis, or breast cancer in general something you wanted/needed to discuss with others?

If so, who did you talk to or want to talk to?

Did you talk with your partner/spouse about the diagnosis or treatment?

If so, what specifically did you talk about?

Describe for me the extent to which you have found this to be helpful to you.

How much have you personally used your immediate family for support?

How much has your immediate family use you for support?
